# Effects and safety of tanreqing injection on viral pneumonia: A protocol for systematic review and meta-analysis: Retraction

**DOI:** 10.1097/MD.0000000000023583

**Published:** 2020-11-25

The article, “Effects and safety of tanreqing injection on viral pneumonia: A protocol for systematic review and meta-analysis”,^[1]^ which published in Volume 99, Issue 37 of *Medicine*, is being retracted due to confirmed scientific misconduct by the authors. The authors admit to fabricating the type of clinical trials included, the selection process of eligible papers, and the method of data extraction and independent data screening. The authors also admit to partially plagiarizing published Medicine article “Effects and safety of tanreqing injection on viral pneumonia: A protocol for systematic review and meta-analysis”.^[2]^
